# Serum Homocystein Level in Patients With Scleroderma

**DOI:** 10.5812/ircmj.3672

**Published:** 2013-01-05

**Authors:** Mohammadali Nazarinia, Mesbah Shams, Eskandar Kamali Sarvestani, Saeede Shenavande, Maryam Khademalhosseini, Zeinab Khademalhosseini

**Affiliations:** 1 Shiraz University of Medical Sciences, Shiraz, IR Iran; 2 Endocrine and metabolism research center, Shiraz University of Medical Sciences, Shiraz, IR Iran; 3 Autoimmune Disease Research Center, Shiraz University of Medical Sciences, Shiraz, IR Iran

**Keywords:** Scleroderma, Systemic, Homocysteine, Autoimmunity, Raynauds Disease

## Abstract

**Background:**

Systemic Sclerosis (SSc) is a systemic connective tissue disease. In this study, we compared the serum Homocystein (Hcy) level between patients with SSc and normal control group.

**Objectives:**

The current study was conducted to determine whether serum Hcy levels are elevated in SSc patients and whether there is any correlation between Hcy levels and RP, Gastro intestinal and lung involvement.

**Patients and Methods:**

Forty one patients who fulfilled the diagnostic criteria for SSc (39 females and 5 males) and Forty four community-based healthy individuals (sex and age matched) were enrolled in to the study. Serum Hcy, vitamin B12, and folate levels were determined.

**Results:**

Thirty three patients (70.45%) had GI involvement, twenty two patients (50%) had lung involvement and twenty seven patients (61.36%) had Raynaud’s phenomena. Mean serum Hcy level in control group was 22.78 ± 6.018 μmol/L and in case group was 19.43 ± 7.205 μmol/L, shows that the serum Hcy level in control group was significantly higher than patients (P = 0.020).

**Conclusions:**

Serum Hcy level is significantly lower in SSc patients than in control group. There is no statistically significant correlation between serum Hcy level and organ involvements.

## 1. Background 

Systemic sclerosis “scleroderma” (SSc) is a disorder of connective tissue affecting skin and internal organs, characterized by fibrotic changes of peripheral and visceral architecture (1). Homocystein a nonessential sulfur-containing amino acid is derived from methionine (2, 3). It is formed during the conversion of methionine to cysteine. Elevations in total plasma Hcy level may be caused by folate and vitamin B12 deficiencies, which are the cofactors for the enzyme reactions involved in Hcy metabolism (4, 5).To date, it has been revealed that multiple rheumatic may be associated with hyper homocysteinemia, which may be associated with cardiovascular events (6-9).

## 2. Objectives

The current study was conducted to determine whether serum Hcy levels are elevated in SSc patients and whether there is any correlation between Hcy levels and RP, Gastro intestinal (1) and lung involvement.

## 3. Patients and Methods

This prospective, case-control study incorporating 44 patients of SSc who fulfilled the diagnostic criteria. Forty four community-based healthy individuals constituted the control group. The exclusion criteria were pregnancy, History of Diabetic mellitus, thyroid disease, chronic disease except SSc and Methotrexate (MTX) consumption. Moreover, blood samples were withdrawn from a peripheral vein after 8 hours of fasting. All the patients were informed about the aims of the study and written consents were obtained from them. The blood samples were centrifuged for 20 minutes at 2000 rpm. The sera were collected and kept at -70°C until assays. The measurement of Hcy level was performed using enzyme-linked immune sorbent assay (ELISA) kit (Axis homocysteine EIA, REF FH CY 100). Moreover, serum vitamin B12 and folate levels were determined by radioimmunoassay (RIA). Serum Hcy levels lower than 15µmol/lit, serum folate levels above 1.5 ng/mL and serum vitamin B12 levels above 160 pg/mL were considered normal. Then the patients were categorized according to the presence or absence of GI, lung involvement and RP and serum Hcy level were compared between them. Moreover, in both groups the correlation between serum levels of Hcy and folate or vitamin B12 was examined. The data were presented as mean ± standard deviation (SD) and analyzed using SPSS software version 10. Student's t-test and correlation tests were used to compare the variables. P ≤ 0.05 were considered statistically significant.

## 4. Results

The patients were consisted of 39 females and 5 males with a mean age of 37.15 ± 12.07 (range: 9-69 years). Mean age of control group was 39.43 ± 10.92 (range: 13 - 70 years) (5 men and 39 women). Thirty three (70.45%), 22 (50%), 27 (61.36%) have gastrointestinal, lung involvement or Raynaud׳s phenomena respectively. Mean serum Hcy level in control group is significantly higher (Table 1). The mean serum Hcy level was high in patients with GI involvement, lung involvement and RP and higher than patients without involvement (Table 2). Our findings suggest that serum Hcy level has no significant correlation with GI involvement (P = 0.831), lung involvement (P = 0.336) and RP (P = 0.778) in patients who are diagnosed as having SSc but we detected higher serum Hcy level in patients with GI, lung involvement and RP than patients without involvement. Serum levels of folate and vitamin B12 were comparable in patients with SSc and control group. During our study we found there is a negative significant correlation between serum Hcy level and serum vitamine B12 and folate levels.

**Table 1:** Mean Serum Levels of Hcy, Folate, and Vitamin B12 in Case and Control groups

Serum	Case group, No. (Mean ± SD)	Control group, No. (Mean ± SD)	P value
** Homocysteine, μmol/L**	44 (19.43 ± 7.20)	44 (22.78 ± 6.01)	.020
** B12, pg/mL**	44 (197.90 ± 60.68)	44 (250.88 ± 108.86)	.006
** Folate, ng/mL**	44 (3.79 ± 1.04)	44 (4.40 ± 1.64)	.041

**Table 2:** Serum Homocystein, Folate and Vitamin B12 in Patients with and Without GI, Lung Involvement and Raynaud’s Phenomena

	Serum Homocystein Level, µmol/l, No. (Mean ± SD)	Serum B12 Level, pg/ml, No. (Mean ± SD)	Serum Folate Level, ng/ml, No. (Mean ± SD)
**GI involvement**			
Yes	31 (19.13 ± 6.99)	31 (197.43 ± 62.76)	31 (3.77 ± 1.14)
No	13 (18.53 ± 7.74)	13 (210.98 ± 40.39)	13 (3.96 ± 0.67)
***P* value**	0.831	0.566	0.654
**Lung involvement**			
Yes	22 (20.86 ± 7.51)	22 (191.36 ± 54.54)	22 (3.68 ± 0.99)
No	22 (17.98 ± 8.18)	22 (226.20 ± 49)	22 (3.93 ± 1.09)
***P* value**	0.336	0.094	0.538
**Raynauds phenomena**			
Yes	17 (19.21 ± 6.88)	17 (191.93 ± 57.56)	17 (3.47 ± 0.84)
No	27 (19.87 ± 7.07)	27 (199.23 ± 61.47)	27 (3.94 ± 1.12)
***P* value**	0.778	0.714	0.178

## 5. Discussion

In this study, we found that serum Hcy level in patients with SSc was higher than normal range but significantly lower than Hcy level in control group. In A Pilot Study of Subclinical Coronary Atherosclerosis in SSc, patients with SSc were found to have significantly higher levels of coronary Hcy than age and sex-matched controls (2). To date, it has been revealed that multiple rheumatic diseases including RA, RP, AS, SLE and gout may be associated with hyperhomocysteinemia, which may be associated with cardiovascular events (6-9). In many studies which focused on serum Hcy level on rheumatic disease serum Hcy level was higher in case group than control group (6, 7, 10, 11) but some studies do not support this finding. Among them, Szamosi et al. could not find significant differences in the Hcy level between SSc patients and controls or between lcSSc or dcSSc subtypes (12). All of these indicated that there is controversy between different reports which have compared serum Hcy level in case and control groups in rheumatic diseases. In our study serum Hcy level was higher in control group than case group and we couldn’t find any logical reason for this. Also we couldn’t find any study in Iran who directly measured serum Hcy level in healthy population. Our findings suggest that serum Hcy level has no significant correlation with these involvements. In previous study Haagsma et al. (13) could not find any correlation between plasma Hcy levels and clinical efficacy in patients with RA. According to previous studies there are higher plasma levels of Hcy in patients with RP (14). Elevated serum Hcy levels were noted for a cohort of SLE patients with RP compared to those not evidencing RP and healthy controls (5). Owing to the shared similar pathogenesis of Hcy-associated vasculopathy and RP and the recent reports indicating that Hcy plays some role in the vascular complications associated with certain collagen diseases (11), these provide the rationale for this study. The association between serum Hcy levels and RP is still controversial (11, 14). The investigators divided 71 patients with SSc into three different groups based on the level of their pulmonary involvement. They evaluated lung involvement, and also determined plasma Hcy concentration in all of the patients and controls. They found Hcy concentration was significantly higher in patients than in controls. It was also linked with severity of lung involvement (10, 11). Also we noted that there is a significant negative correlation between serum Hcy level and serum vitamin B12 and folate level. One limitation of our study was small size sample. The strength of this study was measurement of vitamin B12 and folate along with serum Hcy level which the former influence the metabolism of Hcy. Our study is not able to elucidate the cause and effect relationship between circulating Hcy levels and RP, GI involvement and lung involvement, nor it is able to shed any light on the rationale for the observation that the serum Hcy levels were .We concluded that serum Hcy level is significantly lower in SSc patients than in control group. Our findings suggest that although there is higher serum Hcy level in patients with GI, lung involvement and RP than patients without involvement; there is no statistically significant correlation between serum Hcy level and these involvements in patients who are diagnosed as having SSc. Also according to our results surprisingly mean serum Hcy level in control group was higher than case group that is not matched with results in previous studies and this, needs more investigations in future.
